# Dose-Dependent Responses of Weaned Piglets to Multi-Species Solid-State Fermented Apple Pomace: Enhanced Growth Performance, Intestinal Health, and Gut Microbiota Modulation

**DOI:** 10.3390/ani16020334

**Published:** 2026-01-21

**Authors:** Jiongjie He, Shengyi Wang

**Affiliations:** Key Laboratory of New Animal Drug Project of Gansu Province, Key Laboratory of Veterinary Pharmaceutical Development, Ministry of Agriculture and Rural Affairs, Lanzhou Institute of Husbandry and Pharmaceutical Sciences of Chinese Academy of Agriculture Sciences, Lanzhou 730050, China; ajie7298@163.com

**Keywords:** fermented apple pomace, weaned piglets, gut microbiota, growth performance, intestinal health, solid-state fermentation, dose–response

## Abstract

Background/Objectives: Apple pomace, a major by-product of juice production, represents both an environmental burden and an underutilized resource. This study aimed to enhance the nutritional value of apple pomace via solid-state fermentation (SSF) to develop a functional feed ingredient and systematically evaluate its effects on growth, metabolism, and intestinal health in weaned piglets. Methods: Apple pomace was fermented using a multi-species consortium (*Geotrichum candidum*, *Saccharomyces cerevisiae*, *Rhizopus oryzae*, *Bacillus subtilis*, and *Trichoderma viride*). A total of 180 weaned piglets were fed iso-nitrogenous diets containing 0, 2, 4, 6, 8, or 10% fermented apple pomace for 35 days. Growth performance, serum biochemical and immuno-antioxidant indices, diarrhea incidence, jejunal morphology, and fecal microbiota were analyzed. Results: Dietary fermented apple pomace supplementation showed dose-dependent effects. The 8% fermented apple pomace group exhibited optimal growth performance, with increased average daily gain and feed intake and reduced feed-to-gain ratio (*p* < 0.05). Serum analysis indicated enhanced protein synthesis, antioxidant capacity (T-AOC, SOD, GSH-Px), and immunoglobulin levels (IgA, IgG, IgM), along with reduced urea nitrogen and oxidative stress marker MDA. This group also had the lowest diarrhea rate, associated with improved jejunal villus morphology. Microbiota analysis revealed that 8% fermented apple pomace effectively increased α-diversity, promoted beneficial bacteria (e.g., lactic acid bacteria and butyrate-producing *Clostridium sensu stricto_1*), and suppressed pathogens (*Escherichia coli*, *Salmonella*, *Streptococcus*). Conclusions: Multi-species SSF successively enhanced the nutritional profile of apple pomace. Inclusion at 8% showed the most favorable response in terms of growth performance, metabolic profile, and immune–antioxidant status in weaned piglets, mediated through improved intestinal morphology and targeted modulation of the gut microbiota toward a more diverse and beneficial ecosystem. These findings support the high-value, functional utilization of apple pomace as a feed additive in swine nutrition.

## Simple Summary

Apple juice production generates large amounts of leftover pulp, posing a waste problem. This study aimed to recycle this pulp by fermenting it with a mix of beneficial microbes, turning it into a nutritious feed material. We tested this fermented apple pulp in diets for young, weaned piglets, who often struggle with poor digestion and diarrhea. Adding 8% of this fermented product to their feed led to the best outcomes: piglets grew faster and healthier, had stronger immune systems and better digestion, and experienced far less diarrhea. This improvement was linked to positive changes in their gut bacteria. Our work demonstrates a practical way to transform apple waste into a valuable feed ingredient that supports sustainable pig farming and reduces the environmental burden.

## 1. Introduction

As the world’s largest producer and processor of apples, China generates a massive amount of by-product—apple pomace—alongside its considerable economic output [1]. It is estimated that millions of tons of fresh apple pomace are produced annually in the country [2]. Currently, only a small fraction is utilized in low-value applications, while the majority is discarded or landfilled. Due to its high moisture content and perishable nature, this practice not only represents a significant waste of plant-based nutrients and fiber resources but also contributes to environmental pollution, posing a bottleneck for the sustainable development of the apple industry [3].

From a nutritional perspective, while fresh apple pomace contains certain carbohydrates, minerals, and vitamins, its inherent drawbacks limit its direct use in the feed industry [4]. Its crude protein content is exceptionally low (typically below 5%), which is insufficient to meet animal growth requirements [5]. Moreover, its high levels of anti-nutritional factors, such as pectin and tannins, may interfere with nutrient digestion and absorption [6]. Therefore, efficient biotransformation of apple pomace is crucial to unlocking its feed value and converting this waste into a resource.

Solid-state fermentation (SSF) technology is regarded as an effective approach for enhancing the feed value of agricultural by-products [7]. By inoculating defined microbial strains (e.g., yeasts, molds, lactic acid bacteria) under controlled conditions, SSF can achieve multiple objectives: degrading cell wall structures and anti-nutritional factors to improve substrate digestibility; utilizing the matrix’s carbon and nitrogen sources to synthesize microbial protein, thereby significantly elevating the product’s crude protein and essential amino acid levels; and simultaneously producing organic acids, digestive enzymes, probiotics, and various bioactive metabolites [8]. Previous studies have confirmed that fermenting apple pomace with single or simple consortia (e.g., *Candida utilis*, *Aspergillus niger*) can increase its protein content to varying degrees [9]. However, existing research has largely focused on the fermentation process itself or basic nutritional characterization. Systematic evaluation of products derived from more complex, multi-strain, and optimized fermentation systems—particularly in livestock and poultry, and especially in physiologically sensitive animals—remains insufficient [10]. Studies that delve into the underlying mechanisms from multiple dimensions, such as intestinal health and immunomodulation, are even more limited.

This research gap is particularly significant in the context of feeding weaned piglets. Weaning is a critical stress phase in early life, often accompanied by a sharp decline in feed intake, impaired intestinal barrier function, disruption of gut microbiota, and an immature immune system, ultimately manifesting as growth retardation and high diarrhea incidence [11]. Identifying safe and effective functional feed additives to alleviate weaning stress, maintain intestinal health, and enhance immunity is a major focus in modern swine production and the feed industry [12]. Similarly, dietary optimization during the nursery phase is crucial, as it directly determines growth efficiency and economic returns in the finishing period [13].

Based on the aforementioned context, we hypothesized that dietary inclusion of multi-species fermented apple pomace would exert dose-dependent effects on weaned piglets. Specifically, we postulated that (1) supplementation with fermented apple pomace would enhance growth performance, but this effect would follow a non-linear pattern, with an optimal inclusion level beyond which benefits might plateau or decline; (2) fermented apple pomace would improve intestinal health, as indicated by enhanced jejunal morphology and reduced diarrhea incidence, in a dose-responsive manner; (3) fermented apple pomace would modulate systemic metabolism and enhance immuno-antioxidant status, reflected in serum biochemical and immunological parameters; and (4) these improvements would be mediated, at least in part, through targeted modulation of the gut microbiota, increasing its diversity and favoring the proliferation of beneficial bacterial taxa while suppressing potential pathogens.

Therefore, this study aims to systematically address these issues. We first established an efficient multi-strain (*Geotrichum candidum*, *Saccharomyces cerevisiae*, *Rhizopus* sp., *B. subtilis*, *Trichoderma viride*) synergistic SSF process through multi-round screening and optimization, successfully converting low-value apple pomace into a high-protein feed ingredient. Subsequently, via a rigorously designed animal feeding trial using weaned piglets as a model, we systematically evaluated the effects of this fermented apple pomace on growth performance, nutrient metabolism, serum immune and hormonal indices, gut microbiota structure, and diarrhea incidence. This study not only provides a validated technical solution for the resource utilization of apple pomace but, more importantly, elucidates the potential mechanisms by which it acts as a functional feed additive in alleviating weaning stress, enhancing intestinal barrier function, and improving immunity from an animal physiology and health perspective, thereby offering a solid theoretical and practical foundation for its scientific application.

## 2. Materials and Methods

### 2.1. Preparation of Fermented Apple Pomace

Microbial strains: *Geotrichum candidum*, *Saccharomyces cerevisiae*, *Rhizopus* sp., *B. subtilis*, and *Trichoderma viride* were obtained from the China Center of Industrial Culture Collection (CICC), Beijing, China and the American Type Culture Collection (ATCC), Manassas, VA, USA. These strains were combined into a composite inoculum at a ratio of *G. candidum*:*S. cerevisiae*:*Rhizopus oryzae*:*B. subtilis*:*T. viride* = 2:2:0.5:0.5:1 (dry weight basis).

Fermentation substrate: The substrate consisted of dry apple pomace (81.3%), wheat bran (15%, Zhongliang Grains Group, Zhengzhou, China), urea (1.5%, Sigma-Aldrich, St. Louis, MO, USA), ammonium sulfate (2%, Sinopharm Chemical Reagent Co., Ltd., Shanghai, China), dipotassium phosphate (0.1%, Alfa Aesar, Haverhill, MA, USA), and calcium carbonate (0.1%, Thermo Fisher Scientific, Waltham, MA, USA). The substrate-to-water ratio was maintained at 1:0.8.

Fermentation process: The composite inoculum was added to the substrate at an inoculation rate of 10% (*w*/*w*). Solid-state fermentation was conducted at 31 °C for 72 h in a constant temperature and humidity incubator (Model SPX-150, Shanghai Jinghong Laboratory Equipment Co., Ltd., Shanghai, China). The fermented product was then dried at 65 °C using an air-drying oven (Model DHG-9070A, Shanghai Yiheng Scientific Instrument Co., Ltd., Shanghai, China), ground with a universal high-speed grinder (Model FW-100, Tianjin Taisite Instrument Co., Ltd., Tianjin, China), and stored for subsequent use. This fermentation process significantly increased the crude protein content of the apple pomace itself from 3.81% (raw material) to 5.31%. Due to the low inclusion rates (2% to 10%) of the fermented product in the experimental diets, and the concomitant adjustment of other protein sources (e.g., soybean meal) to maintain isonitrogenous conditions across all dietary treatments, the final analyzed crude protein content of the complete diets ranged from 17.52% to 17.54%.

### 2.2. Animal Trial Design and Management

All animal experimental procedures were approved by the Institutional Animal Care and Use Committee of the Lanzhou Institute of Husbandry and Pharmaceutical Sciences, Chinese Academy of Agricultural Sciences (Approval No.: LIHPS-2018-022; Date: 25 March 2018).

**Figure 1:**
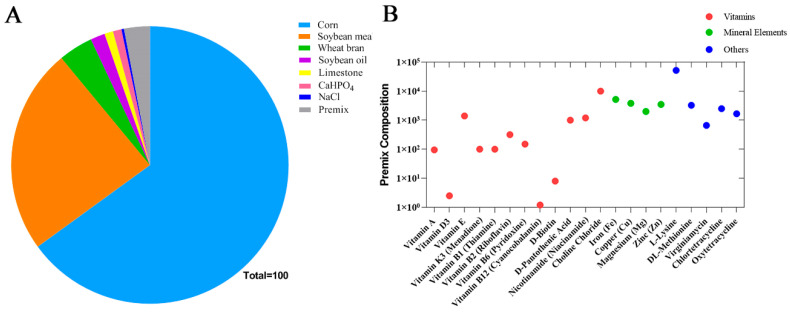
Composition of the basal diet for weaned piglets. (**A**) Percentage composition of nutrients in the basal diet; (**B**) Ingredient composition of the premix per kg of diet.

Trial 1 (Weaned Piglets): A total of 180 healthy weaned piglets ([Duroc × Landrace × Yorkshire]) with an average initial body weight of 7.0 ± 0.5 kg were randomly assigned to one of six dietary treatments. Each treatment consisted of 10 replicates (pens), with 3 piglets per pen. The pen was considered the experimental unit for the assessment of average daily feed intake (ADFI), feed-to-gain ratio (F/G), and diarrhea incidence. Average daily gain (ADG) was calculated based on initial and final individual body weights. The control group was fed a basal diet, while the five treatment groups received iso-nitrogenous diets supplemented with 2%, 4%, 6%, 8%, or 10% fermented apple pomace, respectively. To address the potential variation in metabolizable energy (ME) due to fermented apple pomace inclusion, the diets were formulated to be iso-nitrogenous and iso-energetic. The ME content of fermented apple pomace was estimated based on its analyzed chemical composition (primarily crude protein, ether extract, and nitrogen-free extract) using standard feed energy prediction equations for swine. The inclusion of fermented apple pomace was balanced by proportionally adjusting the levels of corn and soybean meal in the basal diet to maintain a consistent calculated ME concentration across all dietary treatments, ensuring that any observed effects could be attributed to the bioactive components of fermented apple pomace rather than differences in energy intake. The trial lasted for 35 days, including a 3-day adaptation period followed by a 32-day experimental period. The composition and nutritional levels of the basal diet are presented in Figure 1A and Table A1. The composition of the vitamin–mineral premix is detailed in Figure 1B and Table A2. The basal diet was formulated to meet or exceed the nutrient requirements for weaned piglets as recommended by the National Research Council (2012) [14]. The basal diet was formulated to contain 14.18 MJ/kg digestible energy, 17.52% crude protein, 0.77% calcium, 0.43% phosphorus, and 0.90% lysine (Table A3).

### 2.3. Sample Collection

Fecal samples: At the end of the trial, fresh fecal samples were collected from three randomly selected piglets per replicate. A total of 27 composite fecal samples were obtained. Each sample was thoroughly mixed and divided into two portions. One portion was immediately placed into a 50 mL sterile centrifuge tube, transported on ice to the laboratory, and processed promptly for the determination of cultivable fecal microbiota counts. The other portion was stored in a cryogenic tube, flash-frozen on dry ice, and preserved at −80 °C for subsequent analysis of fecal microbial diversity.

Blood samples: On the final morning of the trial, blood samples (5 mL each) were collected from the anterior vena cava of three randomly selected piglets per replicate. Blood was collected into serum tubes, allowed to clot, and then centrifuged at 3000 rpm for 10 min. The resulting serum was separated and stored at −20 °C until analysis.

### 2.4. Measurements and Analytical Methods

Growth performance: Pigs were weighed individually at the beginning and end of the trial on an empty stomach. Feed intake per pen was recorded throughout the experimental period. Average daily gain (ADG), average daily feed intake (ADFI), and the feed-to-gain ratio (F/G) were calculated.

Serum biochemical, immune, and antioxidant indices: Serum levels of total protein, albumin, urea nitrogen, glucose, triglycerides, total cholesterol, triiodothyronine (T3), thyroxine (T4), insulin, and ghrelin were measured using a Hitachi 7020 automatic biochemical analyzer (Hitachi High-Technologies Corporation, Tokyo, Japan). The activities of superoxide dismutase (SOD) and glutathione peroxidase (GSH-Px), as well as T-AOC and malondialdehyde (MDA), were determined using commercial assay kits according to the manufacturer’s instructions (Nanjing Jiancheng Bioengineering Institute, Nanjing, China). Serum concentrations of immunoglobulin G (IgG), immunoglobulin A (IgA), and immunoglobulin M (IgM) were measured using enzyme-linked immunosorbent assay (ELISA) kits (Bethyl Laboratories, Inc., Montgomery, TX, USA).

Diarrhea incidence and cultivable fecal microorganisms (Weaned piglet trial only): The number of piglets with diarrhea in each group was recorded daily throughout the trial. Diarrhea incidence was calculated as follows: (Total number of diarrheal piglet-days)/(Number of piglets × Trial days) × 100%. On day 35, fresh fecal samples were collected from each replicate for microbiological analysis. Total viable counts of specific bacterial groups were determined using the plate count method after 48 h of incubation at 37 °C on selective media: lactic acid bacteria on modified de Man, Rogosa and Sharpe (MRS) agar; *E. coli* on Eosin Methylene Blue (EMB) agar; *Salmonella* on Bismuth Sulfite (BS) agar; and *Staphylococcus aureus* on Baird-Parker agar. Microbial counts were expressed as log10 colony-forming units per gram of feces [log10(CFU/g)].

Fecal microbial community diversity analysis: Total genomic DNA was extracted from fecal samples using the QIAamp Fast DNA Stool Mini Kit (Qiagen, Hilden, Germany) following the manufacturer’s protocol. The concentration and purity of the extracted DNA were assessed. The V3–V4 hypervariable region of the bacterial 16S rRNA gene was amplified by polymerase chain reaction (PCR) using the universal primers 338F (5′-ACTCCTACGGGAGGCAGCA-3′) and 806R (5′-GGACTACHVGGGTWTCTAAT-3′). The PCR mixture (20 μL) contained: 4 μL of 5× FastPfu Buffer, 2 μL of 2.5 mM dNTPs, 0.8 μL each of the forward and reverse primers (5 μM), 0.4 μL of FastPfu Polymerase, 10 ng of DNA template, and ddH_2_O to make up the volume. PCR conditions were as follows: initial denaturation at 95 °C for 3 min; 27 cycles of denaturation at 95 °C for 30 s, annealing at 55 °C for 30 s, and extension at 72 °C for 45 s; followed by a final extension at 72 °C for 10 min. PCR products were checked by 2% agarose gel electrophoresis, purified, and subjected to paired-end sequencing (2 × 300 bp) on an Illumina MiSeq PE300 platform (Illumina, Inc., San Diego, CA, USA) [15]. After quality filtering of the raw sequences, operational taxonomic units (OTUs) were clustered at a 97% similarity threshold using the UPARSE algorithm within the USEARCH software pipeline (version 11.0.667), with chimeric sequences identified and removed during this process. Based on the OTU results, alpha diversity indices, including richness estimators (ACE and Chao1), diversity indices (Shannon and Simpson), and Good’s coverage, were calculated. Taxonomic classification was performed by aligning representative sequences from each OTU against the SILVA 16S rRNA gene reference database (release 138.1) using the RDP Classifier (version 2.13) with a minimum confidence threshold of 0.8. Community structure analysis were performed at different taxonomic levels.

### 2.5. Statistical Analysis

For growth performance (ADFI, F/G) and diarrhea incidence, the pen was used as the experimental unit (*n* = 10). For serum parameters, jejunal morphology, and microbiota data, the individual piglet or sample was used as the experimental unit. Data were initially organized using GraphPad Prism version 9.0.0 (GraphPad Software, San Diego, CA, USA). Statistical analysis was performed using SPSS software version 25.0 (IBM Corp., Armonk, NY, USA). Data were subjected to one-way analysis of variance (ANOVA). When the ANOVA indicated significant differences, means were compared using Duncan’s multiple range test. Differences were considered statistically significant at *p* < 0.05. Results are presented as the mean ± standard error of the mean (SEM).

## 3. Results

### 3.1. Chemical Composition of Experimental Diets

The analyzed chemical composition of the experimental diets confirmed the successful formulation of iso-nitrogenous and iso-energetic diets with graded levels of fermented apple pomace (Table 1). Dry matter content was comparable among all dietary treatments. A concomitant, dose-dependent increase in crude fiber content was observed with higher fermented apple pomace inclusion, with the 8% and 10% fermented apple pomace groups showing significantly elevated levels (*p* < 0.05) compared to the control. This reflects the intrinsic dietary fiber content of the apple pomace substrate, which was preserved or potentially modified in structure during fermentation. Ash content followed a similar pattern, being significantly higher (*p* < 0.05) in the 6%, 8%, and 10% fermented apple pomace groups, aligning with the mineral profile of the fermentation substrate and microbial biomass. No significant differences (*p* > 0.05) were detected for calcium and total phosphorus contents across groups, confirming that the mineral balance of the basal diet was maintained.

### 3.2. Growth Performance Responses to Fermented Apple Pomace Followed a Dose-Dependent Manner, with Beneficial Effects Observed Only at the 8% Inclusion Level

The effects of dietary fermented apple pomace inclusion on growth performance followed a clear dose-dependent pattern, with significant benefits observed exclusively at the 8% inclusion level (*p* < 0.05).

**Figure 2:**
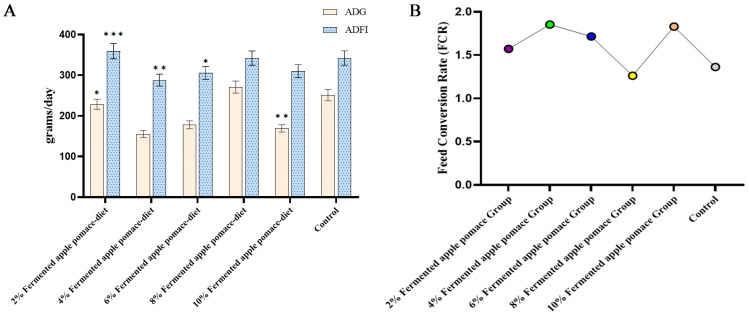
Effects of fermented apple pomace supplementation on growth performance of weaned piglets. (**A**) Average daily feed intake (ADFI) and average daily gain (ADG) in piglets fed isonitrogenous diets with different inclusion levels of fermented apple pomace. (**B**) Feed-to-gain ratio (F/G) of piglets fed the experimental diets, the different colors represent the different supplementation levels of fermented apple pomace. Data are presented as mean ± SD (*n* = 10). * *p* < 0.05, ** *p* < 0.01, *** *p* < 0.001.

As summarized in Figure 2A,B and Supplementary Table S1, compared to the control group, diets containing 2%, 4%, 6%, and 10% fermented apple pomace failed to improve growth performance. Piglets fed these diets either exhibited a significantly lower average daily gain (ADG) or a significantly higher average daily feed intake (ADFI) relative to the control group (*p* < 0.05). Consequently, the feed-to-gain ratio (F/G) was significantly elevated in all these treatment groups (*p* < 0.05).

In contrast, the group receiving the 8% fermented apple pomace-supplemented diet demonstrated optimal outcomes across all key metrics. This group achieved significantly higher ADG and ADFI alongside a significantly lower F/G compared to the control (*p* < 0.05) (Figure 2A,B; Supplementary Table S1). These results indicate that an 8% fermented apple pomace inclusion optimally enhances growth promotion and feed utilization efficiency in weaned piglets.

### 3.3. Dietary Fermented Apple Pomace at 8% Inclusion Synergistically Optimized Systemic Metabolism and Enhanced the Immune–Antioxidant Status in Weaned Piglets

**Figure 3:**
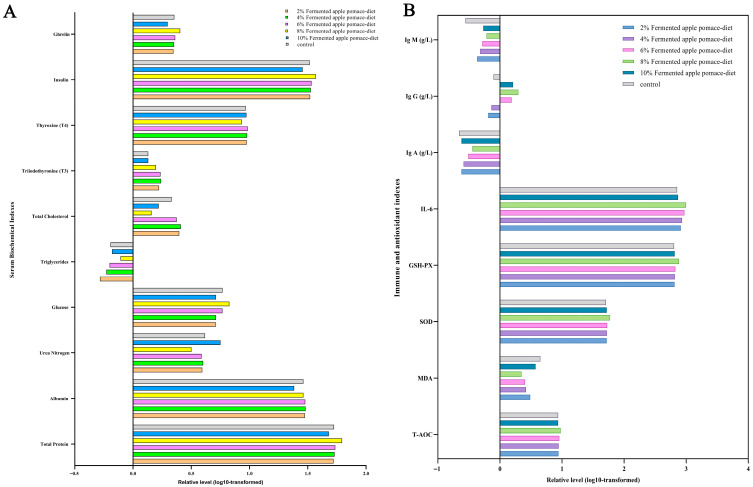
Effects of dietary supplementation with fermented apple pomace on serum physiological and immunological parameters in weaned piglets fed isonitrogenous diets. (**A**) Serum biochemical parameters. (**B**) Serum immunological and antioxidant parameters. Data are presented as mean ± SD.

Dietary inclusion of fermented apple pomace at varying levels induced significant, dose-dependent alterations in serum biochemical parameters in weaned piglets (*p* < 0.05). An inclusion level of 8% fermented apple pomace promoted a distinct and metabolically favorable profile. Specifically, compared to the control and other fermented apple pomace groups, the 8% fermented apple pomace group exhibited significantly elevated serum concentrations of total protein, glucose, insulin, and ghrelin (*p* < 0.05). Concurrently, this group showed the most pronounced reductions in serum urea nitrogen (BUN), total cholesterol, and thyroxine (T4) (*p* < 0.05) (Figure 3A and Supplementary Table S3). Notably, while serum triglyceride levels in the 8% fermented apple pomace group were also significantly lower than in the control (*p* < 0.05), the reduction was less marked than that observed in the 2% and 4% fermented apple pomace groups. Similarly, although the increase in serum triiodothyronine (T3) was not as substantial as in the lower-inclusion groups (2%, 4%, 6%), it remained significantly higher than the control level (*p* < 0.05). A consistent finding across all fermented apple pomace-supplemented groups was a lower serum albumin level compared to the control (*p* < 0.05) (Figure 3A and Supplementary Table S2). However, given that albumin is a key indicator of protein status and hepatic synthesis, this consistent decrease warrants a cautious and contextualized interpretation. It is important to note that the concurrently elevated serum total protein and significantly reduced BUN in the 8% fermented apple pomace group suggest an overall enhancement of protein utilization and nitrogen retention, which may reflect a shift in protein partitioning rather than a simple deficit. Collectively, these coordinated shifts in serum metabolites and hormones are consistent with improvements in metabolic parameters associated with growth. The pattern of increased total protein and insulin alongside decreased BUN suggests enhanced nitrogen utilization for protein synthesis, while the altered lipid and thyroid hormone profiles indicate modulated energy metabolism. This collection of biomarker changes aligns with the lowest F/G ratio observed in this group. The decrease in serum albumin warrants further investigation into long-term protein partitioning.

Systematic modulation of serum immune and antioxidant indices was also observed in response to graded inclusion (2–10%) of fermented apple pomace in isonitrogenous diets. Comprehensive assessment revealed a significantly enhanced antioxidant defense system in all treatment groups compared to the control (*p* < 0.05), manifested by dose-dependent increases in the activities of total antioxidant capacity (T-AOC), superoxide dismutase (SOD), and glutathione peroxidase (GSH-Px), with peak values attained at the 8% inclusion level. Humoral immunity was concurrently activated, as evidenced by significantly elevated circulating levels of immunoglobulins A and M (IgA, IgM) in all FAP groups (*p* < 0.05). Immunoglobulin G (IgG) showed a pattern of initial increase followed by decrease with increasing fermented apple pomace inclusion, also reaching its optimum in the 8% group (*p* < 0.05). Conversely, the concentration of malondialdehyde (MDA), a marker of lipid peroxidation and oxidative damage, was effectively suppressed in all fermented apple pomace groups and reached its lowest level in the 8% group (*p* < 0.05) (Figure 3B and Supplementary Table S3). This synergistic optimization of indices establishes a coherent positive physiological response cascade characterized by “enhanced antioxidant capacity–mitigated oxidative damage–increased immunoglobulin synthesis.”

### 3.4. Optimization of Gut Microbiota and Mucosal Morphology Underlies the Reduction in Diarrhea Incidence by 8% Fermented Apple Pomace in Weaned Piglets

**Figure 4:**
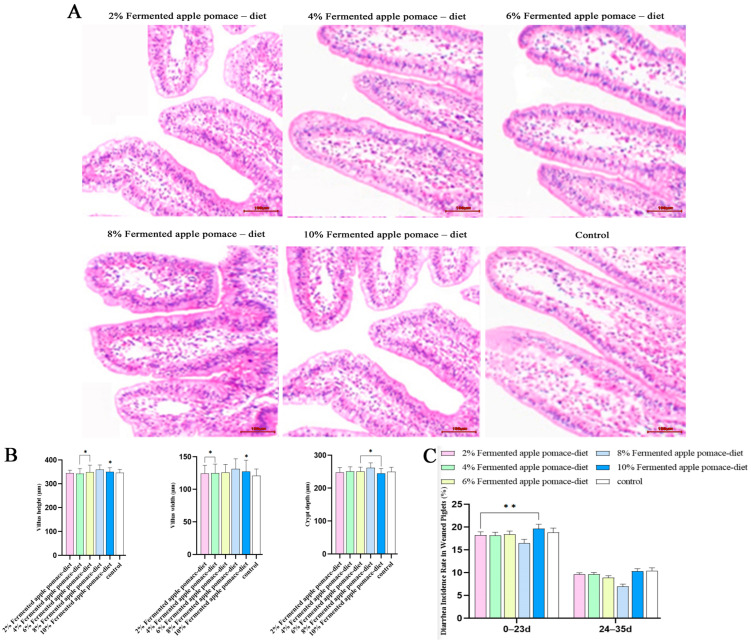
Effects of dietary supplementation with fermented apple pomace on jejunal histomorphometry, jejunum morphometric parameters, and diarrhea incidence in weaned piglets fed iso-nitrogenous diets. (**A**) Representative photomicrographs of jejunal morphology (H&E staining). (**B**) Effects on jejunum villus height, villus width, and crypt depth. (**C**) Diarrhea incidence throughout the trial period. Data are presented as mean ± SD. * *p* < 0.05, ** *p* < 0.01.

Microscopic examination revealed intact jejunal mucosal architecture across all groups. The 8% fermented apple pomace group, however, exhibited superior morphological characteristics compared to the control and other fermented apple pomace groups. Specifically, piglets in the 8% fermented apple pomace group demonstrated more robust and densely packed intestinal villi (Figure 4A). These qualitative observations were substantiated by quantitative morphometric analysis. As detailed in Supplementary Table S4, dietary fermented apple pomace inclusion induced dose-dependent alterations in jejunal villus architecture. Jejunal villus height showed a modest increase in the 6%, 8%, and 10% fermented apple pomace groups compared to the control, with the most pronounced elevation observed at the 8% inclusion level (*p* < 0.05); in contrast, the 2% and 4% groups exhibited a slight decrease. All fermented apple pomace-supplemented groups demonstrated an increase in jejunal villus width relative to the control (*p* < 0.05). Analysis of crypt depth revealed a reduction in the 2% and 10% groups, minimal change in the 6% group, and a slight increase in the 4% and 8% groups, with the greatest depth again recorded in the 8% fermented apple pomace group (*p* < 0.05). These collective findings indicate that the 8% inclusion level most effectively promoted villus development and modulated crypt architecture, thereby expanding the absorptive surface area and providing concrete morphometric evidence for enhanced intestinal digestive and absorptive function.

The incidence of diarrhea in weaned piglets during the 35-day trial exhibited a nonlinear, dose-dependent response to dietary fermented apple pomace inclusion (*p* < 0.05) (Figure 4C and Supplementary Table S5). Diarrhea rates decreased progressively with increasing fermented apple pomace levels up to 8%, where the lowest value was recorded. However, a rebound in diarrhea incidence was observed at the 10% inclusion level. Despite this rebound, all fermented apple pomace-supplemented groups maintained a significantly lower diarrhea rate than the control group (*p* < 0.05). This dose–response pattern suggests an optimal inclusion window for fermented apple pomace, with the 8% level likely conferring the best synergistic benefits for gut health. The significantly optimized jejunal morphology, particularly the increased villus height and the most favorable villus height-to-crypt depth ratio observed in the 8% fermented apple pomace group, provides a direct structural explanation for its lowest diarrhea incidence, supporting a strengthened intestinal barrier and improved nutrient assimilation. The underlying mechanism may involve an optimal combination of dietary fiber, prebiotics, and bioactive compounds in the fermented product, which collectively modulate the gut microbiota structure and fermentation patterns.

### 3.5. 8% Fermented Apple Pomace Modulates Gut Microbiota Homeostasis to Suppress Pathogens and Promote Beneficial Bacteria

**Figure 5:**
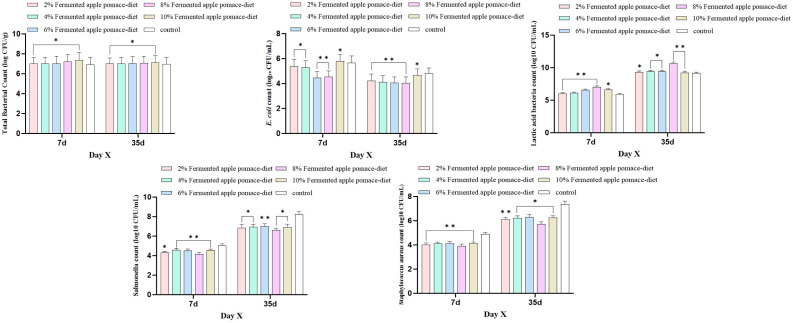
Effects of dietary supplementation with fermented apple pomace on fecal bacterial counts in weaned piglets fed isonitrogenous diets. Quantified bacterial groups include total bacteria, *E. coli*, lactic acid bacteria, Salmonella, and *Staphylococcus aureus*. Data are presented as mean ± SD. * *p* < 0.05, ** *p* < 0.01.

Fresh fecal samples were collected from three randomly selected piglets per group before morning feeding on days 7 and 35 of the trial. For each group, samples were pooled, and 10 g was placed into a sterile tube containing glycerol for preservation at −80 °C until microbial analysis. The total bacterial count remained relatively stable between days 7 and 35 across groups. However, counts in the 8% and 10% fermented apple pomace groups were slightly lower than in other treatment groups, while the control group generally had lower counts than all fermented apple pomace-supplemented groups, with the 8% and 10% groups exhibiting the highest total bacterial numbers. *E. coli* counts changed significantly over time, showing a marked reduction by day 35 in all groups compared to day 7 (*p* < 0.05). On day 7, all fermented apple pomace groups except the 10% group had lower *E. coli* counts than the control, with the 8% group recording the lowest level (*p* < 0.05). By day 35, *E. coli* counts in all fermented apple pomace groups remained significantly lower than in the control (*p* < 0.05), with the 8% group consistently maintaining the lowest count. Lactic acid bacteria (LAB) counts increased significantly from day 7 to day 35 (*p* < 0.05). All fermented apple pomace groups had higher LAB counts than the control at both time points (*p* < 0.05), with the 8% group showing the highest abundance. *Salmonella* and *Staphylococcus aureus* counts followed similar trends, both being higher on day 35 than on day 7. However, the counts of these potential pathogens in all fermented apple pomace groups were lower than those in the control group (*p* < 0.05), with the 8% fermented apple pomace group again showing the lowest levels for both (Figure 5 and Supplementary Table S6).

In summary, dietary supplementation with fermented apple pomace, particularly at the 8% inclusion level, significantly modulated the gut microbiota structure in weaned piglets.

### 3.6. Fermented Apple Pomace Constructs a Beneficial Gut Microecosystem by Enhancing Diversity and Optimizing Microbiota Composition at the Phylum and Genus Levels

**Figure 6:**
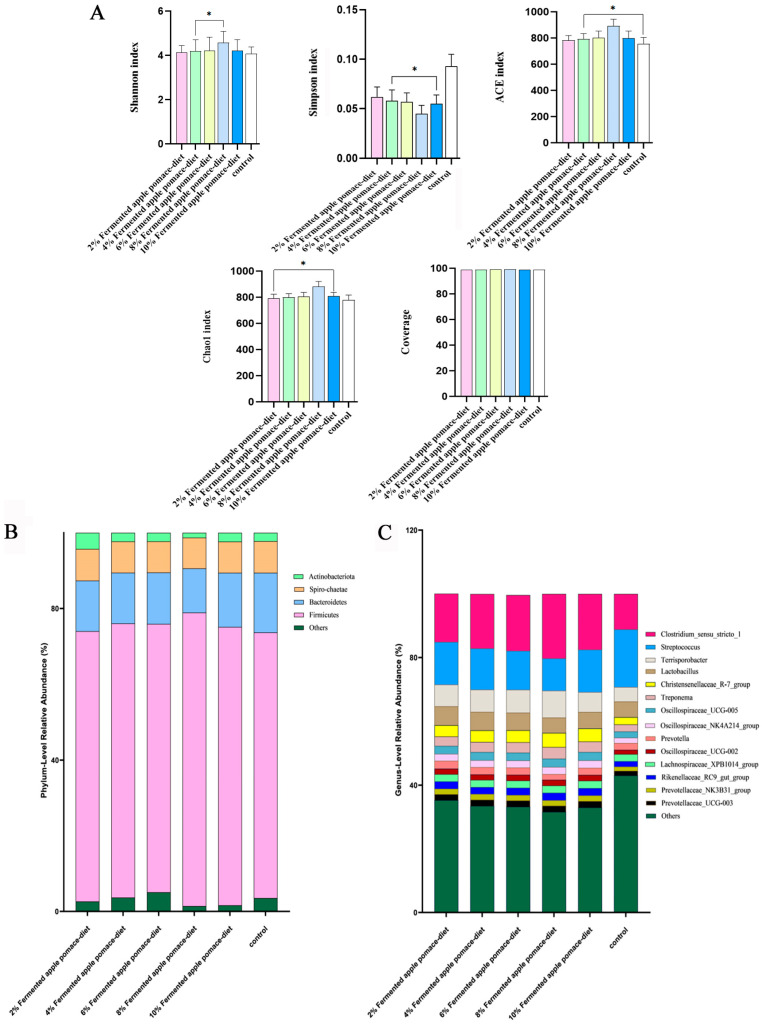
Effects of fermented apple pomace supplementation on the α-diversity and microbial composition at the phylum and genus levels in fecal microbiota of weaned piglets. (**A**) Analysis of α-diversity indices. (**B**) Relative abundance of fecal microbiota at the phylum level. (**C**) Relative abundance of fecal microbiota at the genus level. Data are presented as mean ± SD. * *p* < 0.05.

α diversity analysis revealed that the Coverage values for all samples exceeded 0.99, indicating sufficient sequencing depth to reliably represent the microbial community composition. Compared to the control, all fermented apple pomace-supplemented groups showed consistent alterations in microbial diversity. The Shannon index increased, with the most significant rise observed in the 8% fermented apple pomace group *(p* < 0.05), while the Simpson index significantly decreased, also most markedly in the 8% group *(p* < 0.05). Furthermore, the species richness indices (ACE and Chao1) were significantly higher in all fermented apple pomace groups than in the control *(p* < 0.05), peaking at the 8% inclusion level (Figure 6A and Supplementary Table S7). These results collectively demonstrate that fermented apple pomace supplementation effectively enhanced the diversity and richness of the gut microbial community in weaned piglets, with the 8% inclusion level having the most pronounced effect.

At the phylum level, apart from unclassified taxa, four phyla with a relative abundance greater than 0.1% were identified across groups: Firmicutes, Bacteroidota, Spirochaetota, and Actinobacteria. Compared to the control, all fermented apple pomace groups exhibited an increased relative abundance of Firmicutes, with the 8% group showing the greatest increase (*p* < 0.05). Conversely, the abundance of Bacteroidota decreased in all fermented apple pomace groups, again most significantly in the 8% group (*p* < 0.05). This reciprocal shift in the dominant phyla resulted in an altered community structure at the phylum level. The relative abundance of Spirochaetota showed little variation between fermented apple pomace groups and the control. For Actinobacteria, fermented apple pomace supplementation led to a trend of initial decrease followed by an increase compared to the control, with the highest level in the 2% group and the lowest in the 8% group (Figure 6B and Supplementary Table S8).

Given the high functional heterogeneity within broad taxonomic groups such as phyla, we focused our functional interpretation on changes observed at the genus level. Analysis of 14 core genera (excluding unclassified genera) with a relative abundance greater than 1% revealed that fermented apple pomace supplementation significantly reshaped the gut microbiota structure in a dose-dependent manner, with the most pronounced effects in the 8% group (*p* < 0.05). Key alterations included a significant increase in the relative abundance of the butyrate-producing genus *Clostridium_sensu_stricto_1*, which is known to include butyrate-producing species, across all fermented apple pomace groups, peaking in the 8% group (*p* < 0.05). In contrast, the abundance of the potential pathogen Streptococcus was significantly reduced, with the lowest level found in the 8% group (*p* < 0.05). Furthermore, genera often reported to be associated with fiber degradation and short-chain fatty acid production, such as *Terrisporobacter*, Christensenellaceae_R-7_group, several genera from the Oscillospiraceae family (e.g., UCG-005, NK4A214_group), and genera related to Prevotellaceae (e.g., NK3B31_group, UCG-003), showed increased abundances in fermented apple pomace groups, particularly the 8% group (*p* < 0.05). Notably, the abundance of Lactobacillus exhibited a pattern of initial decrease followed by an increase, with the 8% group showing a relatively lower level (Figure 6C and Supplementary Table S9). This may suggest a potential functional complementarity by other acid-producing bacteria within a more mature microbial community. The lowest abundance of *Prevotella* in the 8% group might indicate a directed shift in carbohydrate fermentation patterns. These taxonomic changes suggest a potential enhancement of microbial metabolic functions, including those linked to butyrogenesis; however, it is important to note that short-chain fatty acid levels were not directly quantified in this study.

## 4. Discussion

This study is grounded in addressing the dual challenges posed by the substantial environmental and resource pressures associated with apple pomace, a major by-product of China’s apple industry, and the intestinal health and growth retardation issues in weaned piglets triggered by weaning stress [16]. While solid-state fermentation technology has emerged as an effective pathway for the high-value utilization of agricultural by-products in recent years, systematic research on the optimization of multi-strain co-fermentation processes for apple pomace and the underlying mechanisms of its impact on the health of weaned piglets remains insufficient [17]. Therefore, this study developed a multi-strain solid-state fermentation process and systematically evaluated the effects of fermented apple pomace on weaned piglets, aiming to provide both theoretical and empirical foundations for the development of functional feed ingredients.

Our findings reveal a nonlinear dose-dependent effect of fermented apple pomace on the growth performance of weaned piglets. Only the 8% inclusion level significantly improved average daily gain and feed intake while reducing the feed-to-gain ratio (*p* < 0.05), indicating the existence of an “optimal inclusion window” [18]. Lower doses may fail to provide sufficient bioactive compounds, whereas excessive inclusion could exert negative effects due to alterations in dietary structure or the accumulation of anti-nutritional factors [19].

The optimal growth performance observed in the 8% fermented apple pomace group was closely associated with its systemic optimization of metabolism and immune function. Piglets in this group exhibited a serum metabolic profile characterized by “high utilization and low catabolism”: increased levels of total protein, glucose, and insulin-like growth factor-1, alongside decreased concentrations of urea nitrogen and total cholesterol (*p* < 0.05), suggesting enhanced protein deposition efficiency and improved energy metabolism [20,21]. Concurrently, significant elevations in serum total antioxidant capacity and the activities of superoxide dismutase and glutathione peroxidase were observed (*p* < 0.05), coupled with a reduction in malondialdehyde content, indicating effective mitigation of systemic oxidative stress [22]. The synchronous increase in immunoglobulin (IgA, IgG, IgM) levels further corroborated its immunoenhancing effect (*p* < 0.05) [23].

The observed systemic benefits are consistent with a model in which localized improvements in intestinal health play a contributory role. Histomorphological analysis demonstrated that the 8% fermented apple pomace group exhibited increased jejunal villus height, decreased crypt depth, and a significantly optimized villus height-to-crypt depth ratio (*p* < 0.05), along with a thicker mucosal glycocalyx layer. These morphological improvements were directly correlated with the lowest incidence of diarrhea in this group (*p* < 0.05) [24,25]. This reinforcement of intestinal structure coincided with a beneficial modulation of the gut microbiota. 16S rRNA sequencing analysis indicated that 8% fermented apple pomace significantly enhanced the alpha diversity of colonic microbiota and altered its compositional structure: the Firmicutes-to-Bacteroidetes ratio increased, and the relative abundance of genera that encompass known SCFA-producers (e.g., *Clostridium sensu stricto_1*) was elevated (*p* < 0.05), while potentially pathogenic genera were suppressed (*p* < 0.05) [26,27]. This suggests that components within fermented apple pomace, such as dietary fiber, could selectively promote the proliferation of beneficial microorganisms. Based on established literature, it is a plausible hypothesis that metabolites from these microbes, particularly butyrate—a primary energy source for colonic epithelial cells—could contribute to the observed improvement in intestinal morphology [28,29]. Thus, a microbiota–SCFA–barrier axis represents one coherent, yet still putative, mechanistic framework to interpret the collective findings of enhanced microbial ecology, intestinal structure, and systemic health. We explicitly note this interpretation is derived from correlative observations in the present study, as direct quantification of SCFAs and molecular markers of barrier function was not performed.

It is important to acknowledge several limitations inherent in the current study, which should be addressed in future research. First, although we hypothesize that SCFAs, especially butyrate, are key metabolites mediating the improvements in gut health, we did not directly measure SCFA concentrations in intestinal contents. This leaves the causal chain of “microbiota alteration–metabolite production–host effect” incomplete [30]. Second, the assessment of intestinal barrier function relied primarily on morphology, lacking quantitative analysis of the gene or protein expression levels of tight junction proteins (e.g., ZO-1, Occludin). This limits the mechanistic understanding of barrier enhancement at the molecular level [31]. Furthermore, while the dietary substitution protocol ensured isonitrogenous conditions, it could not entirely exclude the potential influence of changes in dietary metabolizable energy value resulting from fermented apple pomace inclusion on the observed outcomes.

Beyond the biological mechanisms, the identification of an optimal 8% dietary inclusion level for fermented apple pomace carries direct practical implications for swine production. The significant improvements in growth performance and the marked reduction in diarrhea incidence translate to tangible production benefits, including improved growth rates, enhanced feed efficiency, and lower morbidity. Beyond the biological efficacy, the 8% inclusion level identified in this study represents a practically relevant dosage for potential field applications. Future studies validating these findings under commercial conditions, along with a comprehensive cost–benefit analysis, would be valuable for industry adoption.

In conclusion, this study demonstrates that multi-strain solid-state fermentation can transform apple pomace into a functional feed ingredient that improves the health of weaned piglets. Inclusion at an 8% proportion enhances growth performance by synergistically remodeling the gut microbiota, reinforcing the intestinal physical and immune barrier, and systemically improving host metabolic and antioxidant status. This provides a practical strategy for the valorization of apple pomace and sustainable swine production. Future research should focus on employing metabolomic techniques to quantitatively analyze changes in intestinal and serum metabolite profiles, combined with molecular biology approaches, to elucidate the specific signaling pathways through which key active components in fermented apple pomace regulate host physiology via the “microbiota–gut axis,” thereby fully unraveling its mechanisms of action.

## 5. Conclusions

Solid-state fermentation using a defined multi-species consortium successfully valorized apple pomace into a high-protein feed ingredient. Dietary inclusion of this fermented product at 8% optimized growth performance, protein metabolism, and systemic immune–antioxidant status in weaned piglets (*p* < 0.05). These beneficial effects were associated with improved jejunal morphology, reduced diarrhea incidence (*p* < 0.05), and a targeted modulation of the gut microbiota, which was characterized by enhanced diversity, enrichment of beneficial bacteria (e.g., lactic acid bacteria and butyrate-producing *Clostridium_sensu_stricto_1*), and suppression of pathogenic populations. These findings demonstrate the potential of fermented apple pomace as a functional feed additive to alleviate weaning stress and promote intestinal health in swine production.

## Figures and Tables

**Table 1:** Analyzed chemical composition of the experimental diets (as-fed basis).

Item	Group					
	2% Fermented Apple Pomace Group	4% Fermented Apple Pomace Group	6% Fermented Apple Pomace Group	8% Fermented Apple Pomace Group	10% Fermented Apple Pomace Group	Control Group
Dry matter, %	88.73 ± 1.39 ^a^	88.92 ± 2.46 ^b^	88.87 ± 3.35 ^a^	89.93 ± 2.36 ^a^	89.12 ± 1.94 ^b^	88.24 ± 2.06 ^a^
Crude protein, %	17.53 ± 1.82 ^a^	17.54 ± 1.52 ^a^	17.54 ± 1.04 ^a^	17.53 ± 1.26 ^b^	17.52 ± 1.24 ^b^	17.52 ± 1.64 ^b^
Crude fiber, %	3.89 ± 0.26 ^a^	3.91 ± 0.42 ^a^	3.93 ± 0.18 ^a^	3.95 ± 0.16 ^a^	3.96 ± 0.21 ^b^	3.84 ± 0.26 ^b^
Ash, %	5.31 ± 0.26 ^a^	5.36 ± 0.24 ^a^	5.37 ± 0.26 ^b^	5.46 ± 0.22 ^a^	5.48 ± 0.23 ^b^	5.34 ± 0.23 ^b^
Calcium (Ca), %	0.77 ± 0.03	0.80 ± 0.03	0.81 ± 0.04	0.81 ± 0.04	0.81 ± 0.02	0.77 ± 0.03
Total phosphorus (P), %	0.44 ± 0.02	0.45 ± 0.03	0.45 ± 0.03	0.46 ± 0.02	0.44 ± 0.03	0.43 ± 0.02

Note: Within the same row, means sharing a common superscript letter are not significantly different (*p* > 0.05). Values with different lowercase superscript letters differ significantly (*p* < 0.05).

## Data Availability

The original contributions presented in this study are included in the article/Supplementary Materials. Further inquiries can be directed to the corresponding author.

## Supplementary Materials

The following supporting information can be downloaded at https://www.mdpi.com/article/10.3390/ani16020334/s1: Table S1: Impact of Dietary Fermented Apple Pomace Feed on Weaned Piglet Performance; Table S2: Impact of Dietary Fermented Apple Pomace on Serum Biochemistry of Weaned Piglets; Table S3: Impact of Dietary Fermented Apple Pomace on Immune and Antioxidant Parameters in Growing Pigs; Table S4: Dietary Fermented Apple Pomace and Intestinal Villi in Weaned Piglets; Table S5: Effects of Dietary Fermented Apple Pomace on Diarrhea in Weaned Piglets; Table S6: Impact of Dietary Fermented Apple Pomace on the Fecal Microbiota of Weaned Piglets; Table S7: Analysis of Fecal Microbiota α-Diversity Indices in Weaned Piglets; Table S8: Analysis of Relative Abundance at the Phylum Level in the Fecal Microbiota of Weaned Piglets; Table S9: Analysis of Relative Abundance at the Genus Level in the Fecal Microbiota of Weaned Piglets.

## Abbreviations

The following abbreviations are used in this manuscript:

SSF	solid-state fermentation
T-AOC	the concentration of total antioxidant capacity
SOD	superoxide dismutase
GSH-Px	Glutathione Peroxidase
IgA	Immunoglobulin A
IgG	Immunoglobulin G
IgM	Immunoglobulin M
EMB	Eosin Methylene Blue
BS	Bismuth Sulfite
SEM	standard error of the mean
ADG	average daily gain
ADFI	average daily feed intake
F/G	feed-to-gain ratio
T3	Triiodothyronine
T4	Thyroxine
LAB	Lactic acid bacteria
SCFA	short-chain fatty acid
*E. coli*	*Escherichia coli*
B. subtilis	Bacillus subtilis

## Appendix A

Table A1 provides the formulation of the basal diet for weaned piglets, corresponding to Figure 1A.

**Table A1:** Composition of the Basal Diet.

Ingredient	Percentage (%)
Corn	65
Soybean meal	24
Wheat bran	4
Soybean oil	1.7
Limestone	1
CaHPO_4_	1
NaCl	0.3
Premix	3

**Table A2:** Detailed formulation of the premix, corresponding to Figure 1B.

Ingredient	Dosage	Ingredient	Dosage
Vitamin A	318.50 IU	Vitamin D_3_	100,000.00 IU
Vitamin E	2.10 IU	Vitamin K_3_	100.00 mg
Vitamin B_1_	100.00 mg	Vitamin B_2_	320.00 mg
Vitamin B_6_	150.00 mg	Vitamin B_12_	1.20 mg
D-Biotin	8.00 mg	D-Pantothenic acid	1000.00 mg
Niacinamide	1200.00 mg	Choline chloride	10,000.00 mg
Iron (as Fe)	5200.00 mg	Copper (as Cu)	3833.00 mg
Magnesium (as Mg)	2000.00 mg	Zinc (as Zn)	3500.00 mg
L-Lysine	52,000.00 mg	Virginiamycin	666.70 mg
Methionine	3282.00 mg	Chlortetracycline	2500.00 mg
Oxytetracycline	1667.00 mg		

**Table A3:** Formulated nutrient levels of the basal diet (as-fed basis).

Nutrient	Content
Digestible energy	14.18 MJ/kg
Crude protein	17.52%
Calcium (Ca)	0.77%
Phosphorus (P)	0.43%
Lysine	0.90%
