# Supplementary material for: Dose-Dependent Responses of Weaned Piglets to Multi-Species Solid-State Fermented Apple Pomace: Enhanced Growth Performance, Intestinal Health, and Gut Microbiota Modulation
Source: Animals (Basel). 2026 Jan 21;16(2):334. doi: 10.3390/ani16020334 (PMC12837632; doi:10.3390/ani16020334)
Supplement: Supplementary file 1 [file animals-16-00334-s001.zip › animals-4077843-supplementary.pdf]

Table S1. Impact of Dietary Fermented Apple Pomace Feed on Weaned Piglet Performance

G-fermented apple pomace-diets	Number of piglets	ADG	ADFI	FCR
2% Fermented apple pomace-diet	10	228.63±12.58[a]	359.3±18.53[b]	1.57±0.12[a]
4% Fermented apple pomace-diet	10	155.42±8.54[b]	287.9±14.83[a]	1.85±0.14[a]
6% Fermented apple pomace-diet	10	178.34±9.83[b]	305.5±15.75[a]	1.72±0.13[a]
8% Fermented apple pomace-diet	10	270.93±14.94[b]	342.0±17.61[b]	1.26±0.10[a]
10% Fermented apple pomace-diet	10	169.41±9.32[a]	310.0±15.93[b]	1.83±0.14[a]
Controlt	10	251.13±13.81[b]	342.3±17.69[b]	1.36±0.10[b]

Note: Within the same row, means sharing a common superscript letter are not significantly different (P > 0.05). Values with different lowercase superscript letters differ significantly (P < 0.05), and those with different uppercase superscript letters differ highly significantly (P < 0.01).

Table S2. Impact of Dietary Fermented Apple Pomace on Serum Biochemistry of Weaned Piglets

Serum Biochemical Indexes	Group					
	2% Fermented apple pomace-diet	4% Fermented apple pomace-diet	6% Fermented apple pomace-diet	8% Fermented apple pomace-diet	10% Fermented apple pomace-diet	control
Total Protein (g/L)	52.57±3.25[b]	53.66±3.16[a]	54.45±2.94[a]	62.26±3.99[b]	47.89±3.67[a]	53.13±3.57[b]
albumin (g/L)	29.89±1.35[a]	30.27±1.78[a]	30.09±2.02[b]	29.00±1.94[b]	24.15±1.57[a]	29.00±2.02[b]
Urea Nitrogen (mmol/L)	3.93±0.41[a]	4.00±0.36[a]	3.87±0.33[b]	3.17±0.45[b]	5.63±0.43[b]	4.15±0.38[a]
Glucose (mmol/L)	5.13±0.32[a]	5.16±0.35[b]	5.85±0.32[a]	6.71±0.41[a]	5.16±0.38[b]	5.86±0.36[a]
Triglycerides (mmol/L)	0.52±0.04[b]	0.59±0.04[a]	0.63±0.05[a]	078±0.03[b]	0.66±0.03[a]	0.64±0.03[b]
Total Cholesterol (mmol/L)	2.49±0.25[a]	2.57±0.27[a]	2.37±0.31[b]	1.44±0.21[a]	1.66±0.23[a]	2.14±0.22[a]

Triiodothyronine（T3）(ng/mL)	1.67±0.21^{b}	1.74±0.18^{a}	1.72±0.16^{a}	1.57±0.16^{a}	1.35±0.14^{b}	1.35±0.16^{b}
Thyroxine（T4）(μg/dL)	9.43±1.03^{a}	9.53±0.99^{a}	9.64±1.02^{a}	8.6±0.99^{b}	9.40±0.99^{a}	9.28±1.13^{b}
Insulin (μU/mL)	33.13±2.56^{a}	33.55±2.05^{b}	34.01±2.58^{b}	37.10±2.99^{b}	28.56±2.63^{a}	32.83±3.01^{a}
Ghrelin (ng/mL)	2.22±0.15^{b}	2.25±0.13^{a}	2.30±0.17^{b}	2.54±0.15^{b}	1.98±0.12^{a}	2.26±0.16^{a}

Note: Within the same row, means sharing a common superscript letter are not significantly different (P > 0.05). Values with different lowercase superscript letters differ significantly (P < 0.05), and those with different uppercase superscript letters differ highly significantly (P < 0.01).

Table S3. Impact of Dietary Fermented Apple Pomace on Immune and Antioxidant Parameters in Growing Pigs.

**Immune and antioxidant indexes**	**Group**					
	2% Fermented apple pomace-diet	4% Fermented apple pomace-diet	6% Fermented apple pomace-diet	8% Fermented apple pomace-diet	10% Fermented apple pomace-diet	control
AOC (U/mL)	5.50±0.32^{b}	5.57±0.30^{b}	5.82±0.36^{a}	6.54±0.33^{a}	5.43±0.41^{b}	6.21±0.29^{a}
MDA (nmol/mL)	3.28±0.30^{a}	3.07±0.28^{a}	2.99±0.26^{b}	2.59±0.26^{A}	4.09±0.27^{b}	3.88±0.29^{b}
SOD (U/mL)	38.48±4.52^{b}	38.68±3.95^{a}	39.15±3.84^{b}	43.77±3.95^{a}	38.63±3.56^{a}	42.57±3.39^{b}
GSH-P (U/mL)	550.75±42.75^{a}	553.67±51.24^{b}	558.19±49.95^{a}	601.71±53.27^{A}	553.05±49.26^{b}	579.92±49.27^{a}
IL-6(ng/mL)	694.43±53.47^{a}	708.87±55.69^{b}	740.02±59.36^{a}	766.97±61.37^{b}	665.03±55.27^{a}	714.57±60.36^{a}
Ig A (g/L)	0.81±0.11^{b}	0.85±0.09^{a}	0.98±0.09^{b}	1.09±0.08^{a}	0.81±0.09^{a}	0.71±0.07^{b}
LgG(g/L)	75.10±7.38^{a}	81.36±7.28^{a}	143.29±9.33^{A}	184.47±11.56^{b}	155.71±10.47^{a}	47.21±8.36^{b}

Note: Within the same row, means sharing a common superscript letter are not significantly different (P > 0.05). Values with different lowercase superscript letters differ significantly (P < 0.05), and those with different uppercase superscript letters differ highly significantly (P < 0.01).

Table S4. Dietary Fermented Apple Pomace and Intestinal Villi in Weaned Piglets

Item (Jejunum)	Group					
	2% Fermented apple pomace-diet	4% Fermented apple pomace-diet	6% Fermented apple pomace-diet	8% Fermented apple pomace-diet	10% Fermented apple pomace-diet	control
Villus height (μm)	346.2 ± 11.2^{b}	342.7 ± 21.6^{a}	349.5 ± 29.4^{a}	359.7 ± 19.9^{b}	351.2 ± 16.7^{a}	347.3 ± 13.8^{b}
Villus width (μm)	124.6 ± 12.5^{a}	125.1 ± 13.8^{a}	126.1 ± 12.2^{b}	131.4 ± 15.7^{b}	127.6 ± 17.3^{a}	120.9 ± 10.5^{b}
Crypt depth (μm)	248.7 ± 14.1^{a}	251.6 ± 13.6^{a}	250.9 ± 13.3^{b}	262.3 ± 14.9^{b}	245.7 ± 14.3^{b}	250.3 ± 13.5^{a}

Note: Within the same row, means sharing a common superscript letter are not significantly different (P > 0.05). Values with different lowercase superscript letters differ significantly (P < 0.05).

Table S5. Effects of Dietary Fermented Apple Pomace on Diarrhea in Weaned Piglets

Time	Group					
	2% Fermented apple pomace Group	4% Fermented apple pomace Group	6% Fermented apple pomaceGroup	8% Fermented apple pomace Group	10% Fermented apple pomace Group	Control Group
0—23d	18.25 ± 0.74^{A}	18.16 ± 0.73^{A}	18.45 ± 0.71^{A}	16.52 ± 0.83^{A}	19.68 ± 0.97^{A}	18.86 ± 0.95^{a}
24-35d	9.65±0.37	9.68±0.38	8.94±0.42	7.02±0.49	10.33±0.58	10.46±0.63

Note: Within the same row, means sharing a common superscript letter are not significantly different (P > 0.05) and those with different uppercase superscript letters differ highly significantly (P < 0.01).

Table S6. Impact of Dietary Fermented Apple Pomace on the Fecal Microbiota of Weaned Piglets

Time	Type of microbiota	Group					
		2% Fermented apple pomace Group	4% Fermented apple pomace Group	6% Fermented apple pomace Group	8% Fermented apple pomace Group	10% Fermented apple pomace Group	Control Group
7d	Total bacteria	7.03 ± 0.63^{a}	7.03 ± 0.65^{a}	7.05 ± 0.74^{a}	7.21 ± 0.75^{a}	7.35 ± 0.79^{a}	6.94 ± 0.73^{b}
	Escherichia coli	5.39 ± 0.57^{a}	5.29 ± 0.55^{a}	4.48 ± 0.49^{A}	4.54 ± 0.48^{A}	5.80 ± 0.53^{a}	5.67 ± 0.55^{b}
	Lactic acid	6.05 ± 0.14^{A}	6.13 ± 0.11^{A}	6.57 ± 0.15^{A}	7.03 ± 0.19^{A}	6.63 ± 0.15^{a}	5.92 ± 0.13^{b}

	bacteria						
	Salmonella	4.31±0.13^{a}	4.59±0.15^{A}	4.52±0.17^{A}	4.15±0.18^{A}	4.57±0.11^{A}	5.060±.15
	Staphylococcus aureus	4.03±0.13^{A}	4.12±0.11^{A}	4.13±0.17^{A}	3.92±0.15^{A}	4.15±0.09^{A}	4.89±0.15^{a}
35d	Total bacteria	7.01±0.58^{a}	7.02±0.64^{a}	7.05±0.69^{a}	7.09±0.68^{a}	7.11±0.73^{a}	6.99±0.68^{b}
	Escherichia coli	4.23±0.53^{A}	4.13±0.52^{A}	4.08±0.45^{A}	4.06±0.48^{A}	4.69±0.49^{a}	4.81±0.44^{b}
	Lactic acid bacteria	9.36±0.15^{b}	9.42±0.13^{a}	9.46±0.15^{a}	10.67±0.21^{A}	9.25±0.19^{A}	9.18±0.14^{b}
	Salmonella	6.87±0.34^{a}	6.96±0.24^{a}	7.03±0.25^{A}	6.61±0.18^{A}	6.9±30.29^{a}	8.26±0.24
	Staphylococcus aureus	6.13±0.15^{A}	6.24±0.16^{b}	6.31±0.22^{b}	5.72±0.19^{b}	6.2±70.15^{b}	7.39±0.23^{a}

Note: Within the same row, means sharing a common superscript letter are not significantly different (P > 0.05). Values with different lowercase superscript letters differ significantly (P < 0.05), and those with different uppercase superscript letters differ highly significantly (P < 0.01).

Table S7. Analysis of Fecal Microbiota α-Diversity Indices in Weaned Piglets

**Items**	**Group**					
	2% Fermented apple pomace Group	4% Fermented apple pomace Group	6% Fermented apple pomace Group	8% Fermented apple pomace Group	10% Fermented apple pomace Group	Control Group
Shannon index	4.15±0.3^{a}	4.21±0.5^{b}	4.23±0.6^{b}	4.59±0.5^{b}	4.22±0.5^{a}	4.09±0.3^{a}
Simpson index	0.065±0.010^{b}	0.062±0.009^{a}	0.059±0.010^{a}	0.042±0.009^{a}	0.052±0.010^{a}	0.093±0.011^{b}
ACE index	784.32±6.26^{a}	792.57±42.78^{a}	803.65±51.32^{a}	893.74±50.69^{a}	801.29±53.12^{a}	758.46±46.89^{b}
Chao1 index	793.69±31.84^{a}	799.52±29.64^{a}	807.39±31.26^{a}	883.62±38.93^{a}	812.09±26.43^{a}	781.47±36.89^{b}
Coverage	99.2	99.2	99.3	99.3	99.2	99.1

Note: Within the same row, means sharing a common superscript letter are not significantly different (P > 0.05). Values with different lowercase superscript letters differ significantly (P < 0.05).

Table S8. Analysis of Relative Abundance at the Phylum Level in the Fecal Microbiota of Weaned Piglets

**Microbiota composition at the phylum level**	**Group**					
	2% Fermented apple pomace Group	4% Fermented apple pomace Group	6% Fermented apple pomace Group	8% Fermented apple pomace Group	10% Fermented apple pomace Group	Control Group
Actinobacteriota	4.27%	2.28%	2.27%	1.28%	2.34%	2.22%
Spiro-chaetae	8.36%	8.25%	8.22%	8.13%	8.21%	8.34%
Bacteroidetes	13.37%	13.43%	13.57%	11.69%	14.33%	15.78%

Firmicutes	71.35%	72.35%	70.86%	77.46%	73.49%	70.11%
Others	2.65%	3.69%	5.08%	1.44%	1.63%	3.55%

Table S9. Analysis of Relative Abundance at the Genus Level in the Fecal Microbiota of Weaned Piglets

**Genus composition at the phylum level**	**Group**					
	2% Fermented apple pomace Group	4% Fermented apple pomace Group	6% Fermented apple pomace Group	8% Fermented apple pomace Group	10% Fermented apple pomace Group	Control Group
Clostridium_sensu_stricto_1	15.17%	17.07%	17.52%	20.27%	17.49%	11.14%
Streptococcus	13.38%	12.88%	12.13%	10.04%	13.26%	18.03%
Terrisporobacter	6.77%	7.00%	7.21%	8.45%	6.255	4.54%
Lactobacillus	5.90%	5.78%	5.52%	4.82%	5.175	4.92%
Christensenellaceae_R-7_group	3.54%	3.64%	3.75%	4.43%	4.11%	2.26%
Treponema	2.98%	3.16%	3.27%	3.65%	3.28%	2.23%
Oscillospiraceae_UCG-005	2.49%	2.59%	2.47%	2.60%	2.64%	1.93%
Oscillospiraceae_NK4A214_group	2.20%	2.17%	2.25%	2.28%	2.34%	1.73%
Prevotella	2.45%	2.28%	2.23%	1.74%	2.19%	2.11%
Oscillospiraceae_UCG-002	1.72%	1.7%	1.81%	1.81%	1.835	1.33%
Lachnospiraceae_XPB1014_group	2.27%	2.28%	2.27%	2.28%	2.34%	2.22%
Rikenellaceae_RC9_gut_group	2.31%	2.16%	2.27%	2.34%	2.3%	1.72%
Prevotellaceae_NK3B31_group	1.72%	1.81%	1.74%	1.74%	1.77%	1.3%
Prevotellaceae_UCG-003	1.87%	1.92%	1.94%	1.91%	2.00%	1.46%
Others	35.315	33.52%	33.26%	31.64%	33.02%	43.08%
